# Multi-Modal Detection and Mapping of Static and Dynamic Obstacles in Agriculture for Process Evaluation

**DOI:** 10.3389/frobt.2018.00028

**Published:** 2018-03-27

**Authors:** Timo Korthals, Mikkel Kragh, Peter Christiansen, Henrik Karstoft, Rasmus N. Jørgensen, Ulrich Rückert

**Affiliations:** ^1^Cognitronics & Sensor Systems, Bielefeld University, Bielefeld, Germany; ^2^Department of Engineering, Aarhus University, Aarhus, Denmark

**Keywords:** occupancy grid maps, mapping and localization, obstacle detection, precision agriculture, sensor fusion, multi-modal perception, inverse sensor models, process evaluation

## Abstract

Today, agricultural vehicles are available that can automatically perform tasks such as weed detection and spraying, mowing, and sowing while being steered automatically. However, for such systems to be fully autonomous and self-driven, not only their specific agricultural tasks must be automated. An accurate and robust perception system automatically detecting and avoiding all obstacles must also be realized to ensure safety of humans, animals, and other surroundings. In this paper, we present a multi-modal obstacle and environment detection and recognition approach for process evaluation in agricultural fields. The proposed pipeline detects and maps static and dynamic obstacles globally, while providing process-relevant information along the traversed trajectory. Detection algorithms are introduced for a variety of sensor technologies, including range sensors (lidar and radar) and cameras (stereo and thermal). Detection information is mapped globally into semantical occupancy grid maps and fused across all sensors with late fusion, resulting in accurate traversability assessment and semantical mapping of process-relevant categories (e.g., crop, ground, and obstacles). Finally, a decoding step uses a Hidden Markov model to extract relevant process-specific parameters along the trajectory of the vehicle, thus informing a potential control system of unexpected structures in the planned path. The method is evaluated on a public dataset for multi-modal obstacle detection in agricultural fields. Results show that a combination of multiple sensor modalities increases detection performance and that different fusion strategies must be applied between algorithms detecting similar and dissimilar classes.

## 1 Introduction

In recent years, autonomous robots and systems have influenced the automation of various agricultural tasks. Numerous scientific approaches have shown that adapting robotic advances can improve workflow, minimize manual labor, and optimize yield. Today, however, conventional scenarios still have the human operator in a centralized position of the farming process, supported by various non-centralized controls units. Due to the global trend in automation, the operator will evidently become an observer in upcoming farming scenarios and to a greater extent manage than operate the process. One key aspect of reaching this goal is to ensure safe operation of driverless systems by perceiving the environment from which potential obstacles are detected and avoided. No sensor can single-handedly guarantee this safety in diverse agricultural environments, and, thus, a heterogeneous and redundant set of perception sensors and algorithms are needed for this purpose.

Contrary to self-driving cars whose primary purpose is to travel from A to B, an autonomous farming vehicle must also process the traversed area along its way. Common agricultural tasks are harvesting, mowing, pruning, seeding, and spraying. For these tasks, a simple representation of the environment into traversable and non-traversable areas is insufficient. Instead, an agricultural vehicle requires a distinction between, e.g., traversable areas, such as road and soil, and processable areas, such as grass, crops, and plants. Therefore, obstacle detection in an agricultural context does not simplify to purely identifying objects that protrude from the ground. High grass or crop may appear non-traversable while actually being processable, whereas flat obstacles such as plant seedlings may appear traversable while being non-traversable. A need, therefore, exists for a system that can detect and recognize a large variety of object categories, while at the same time combine the extensive and perhaps unmanageable amount of information into process-specific parameters relevant for either the driver or an autonomous controller.

This paper presents a multi-modal obstacle and environment detection and recognition approach for process evaluation in agricultural fields. The proposed architecture describes a perception pipeline from data acquisition to classification of process-relevant properties along the vehicle path. Detection algorithms are presented for lidar, radar, stereo camera, and thermal camera, individually. Information from all detections is mapped into a global 2D grid-based representation of the environment and fused across object categories, detection algorithms, and sensor modalities. Finally, relevant properties for processing the field such as traversability and yield information along planned trajectories are decoded. The proposed method is evaluated on a public grass mowing dataset recorded in Lem, Denmark, October 2016. The dataset includes both static and dynamic (moving) obstacles, such as humans, vehicles, vegetation, barrels, and buildings as well as structures in the environment such as the grass field and roads.

To the knowledge of the authors, no similar architectures or baselines targeting agricultural applications have previously been published. The proposed architecture, therefore, represents a novel set of procedures to perform acquisition, detection, fusion, mapping, and process evaluation in a multi-modal setup for an unstructured environment in agriculture. As such, the contributions of the paper are as follows:

- An architecture for multi-modal obstacle and environment detection covering detection algorithms, mapping, fusion across sensors and object classes, and path decoding.
- A process evaluation method combining mapped environment detections over time into agriculturally relevant properties using a Hidden Markov model.
- An evaluation on a public agricultural dataset, including lidar, radar, stereo camera, and thermal camera sensor data recorded during grass mowing.

The authors’ approach extends agricultural technology without replacing current work habits, and allows incorporation of state-of-the-art algorithms for comprehensive environment detection and recognition via an efficient mapping approach. Furthermore, it allows for easy changeability and extendability, which is needed in a daily agricultural scenario. In comparison to model-based or parametrized approaches, the non-parametric two-dimensional occupancy grid mapping has more desirable properties for agricultural scenarios, where mainly the vegetated area is of interest. Analytical solutions as well as relevant heuristics have been applied to build the inverse sensor models (ISM) which incorporate the sensor information as well as its localization.

**Figure 1:**
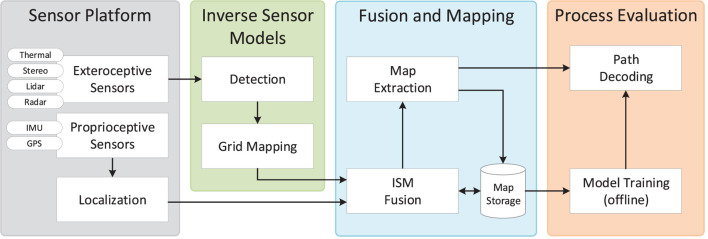
System architecture including information flow.

The proposed architecture is depicted in Figure 1. A sensor platform is mounted on a tractor traversing a field along a preplanned trajectory. A number of exteroceptive sensors collect synchronized perception data used for object detection, whereas proprioceptive sensors are used for global localization of the vehicle. For each sensor modality, an inverse sensor model (ISM) includes an algorithm for detecting a number of object categories (e.g., *human*, *vegetation*, and *building*) and a mapping to align detection information from various algorithms using a 2D occupancy grid map (OGM) representation in the local sensor frame. Detection algorithms include deep learning methods for object detection, semantic segmentation, and anomaly detection on color images, dynamic thresholding on thermal images, point-wise feature extraction and classification of lidar point clouds, and tracking of radar detections. In the fusion and mapping step, OGMs for all sensors and object categories are first localized globally and then updated temporally with the occupancy grid map algorithm by late fusion on a decision level. Finally, they are fused spatially to extract a global map of the environment. We present both binary (occupied/unoccupied) and semantical (object category-specific) maps, allowing further processing in subsequent algorithms. A final decoding step operates on the fused semantical maps and applies a Hidden Markov model to extract relevant process-specific parameters (e.g., harvesting, mowing, or weed-spraying) along the predefined trajectory of the vehicle. The final output could be used to alert a driver with human-understandable information, or directly by a control system for completely autonomous operation.

The paper is divided into six sections. Section 2 introduces related work on obstacle detection in agricultural applications. Section 3 presents the proposed method consisting of each of the four building blocks from Figure 1. Section 4 presents the experimental dataset and results for static and dynamic obstacle and environment detection as well as decoding of process-relevant parameters. Section 5 provides a discussion of the overall approach, while Section 6 concludes the paper and suggests future work.

## 2 Related Work

Robotic automation is emerging for numerous agricultural tasks. The main objective is to reduce production costs and manual labor, while increasing yield and raising product quality (Luettel et al., 2012; Bechar and Vigneault, 2017). A significant milestone is to make robots navigate autonomously in dynamic, rough, and unstructured environments, such as agricultural fields or orchards. To some extent, this has been possible for around two decades with automated steering systems utilizing global navigation systems (Abidine et al., 2004). To eliminate the need for a human operator, however, strict safety precautions are required including accurate and robust risk detection and obstacle avoidance.

Today, only small robots are commercially available that incorporate obstacle avoidance and operate fully autonomously in various agricultural domains (Harvest Automation, 2012; Lely, 2016). Commercialized self-driving tractors or harvesters, however, currently only exist as R&D projects (ASI, 2016; Case, 2016; Kubota, 2017).

In scientific research, the concept of an autonomous farming vehicle with obstacle avoidance dates back to 1997 where a camera was used as an anomaly detector to identify structures different from crop (Ollis and Stentz, 1997). Since then, several systems have been proposed for detecting and avoiding obstacles (Cho and Lee, 2000; Stentz et al., 2002; Griepentrog et al., 2009; Moorehead et al., 2012; Emmi et al., 2014; Ball et al., 2016).

A simplified representation of the environment into traversable and non-traversable regions is common for autonomous navigation (Papadakis, 2013). A path may be non-traversable if it is blocked by obstacles, or if the terrain is too rough or steep. Similarly, anomaly or novelty detection is used to find anything that does not comply with normal appearance and is, thus, used to detect obstacles (Sofman et al., 2010; Ross et al., 2015; Christiansen et al., 2016a). However, for many agricultural tasks, such as harvesting, mowing, and weed spraying, further distinction between obstacles and traversable vegetation is necessary. In one application, apparent obstacles such as crops or high grass may be traversable, whereas in another, small plants at ground level may represent obstacles and thus be non-traversable. Distinction into object, vegetation, and ground is common (Wellington and Stentz, 2004; Lalonde et al., 2006; Bradley et al., 2007; Kragh et al., 2015), whereas a few approaches explicitly recognize classes such as humans, vehicles and buildings (Yang and Noguchi, 2012; Christiansen et al., 2016b).

In the literature, obstacle detection systems often rely on a single sensor modality (Rovira-Mas et al., 2005; Reina and Milella, 2012; Fleischmann and Berns, 2015). These systems, however, are easily affected by varying weather and lighting conditions and, thus, present single points of failure. Christiansen et al. (2017) discusses advantages and disadvantages of various sensor technologies. For instance, a color camera captures visual information similar to humans and can be used to recognize visually distinctive objects. Similarly, a thermal camera captures heat radiation and can distinguish living obstacles such as humans and animals from the background. However, cameras in general are unable to reliably detect object positions and are easily interfered by direct sunlight and changes in weather conditions. On the other hand, lidar and radar sensors are robust to varying weather and lighting conditions and recognize structural differences with high precision. However, the lack of visual information only allows for a few distinguishable object classes. Therefore, a safety system must have a heterogeneous and complementary sensor suite with multiple sensing modalities that have an overlapping frustum^1^ and complement each other in terms of detection capabilities and robustness. Sensor fusion is the concept of combining information from multiple sources to reduce uncertainty in locality and class affiliation. Early fusion combines raw data from different sensors, whereas late fusion integrates information at decision level. In both cases, sensor data need to be compatible.

Lidar, radar, and stereo cameras are all range sensors operating in the domain of metric 3D coordinates. Lidar and radar have been fused with early fusion using a joint extrinsic calibration procedure (Underwood et al., 2010) and with late fusion for augmented traversability assessment (Ahtiainen et al., 2015). Similarly, lidar and stereo camera have been fused with late fusion for traversability assessment (Reina et al., 2016). Often, a grid-based representation such as occupancy grid maps (Elfes, 1990) is used, allowing simple probabilistic fusion and subsequent path planning on the late fused decision level. Monocular cameras operate in the domain of non-metric pixels and can be fused directly under assumption of negligible parallax errors. Examples are available of color and thermal camera fusion for object detection using both early (Davis and Sharma, 2007) and late (Apatean et al., 2010) fusion.

Fusion across domains is possible only when a transformation between them exists. By projecting 3D points onto corresponding 2D images, range sensors can be fused with cameras. With this approach, lidar and color cameras have been combined for semantic segmentation and object recognition using both early (Dima et al., 2004; Wellington et al., 2005; Häselich et al., 2013) and late (Laible et al., 2013; Kragh and Underwood, 2017) fusion. Similarly, image data in pixel-space have been transformed to metric 3D coordinates with inverse perspective mapping (Bertozzi and Broggi, 1998; Konrad et al., 2012). Here, a ground plane assumption is used to invert the perspective effect applied during image acquisition, such that image data are compatible with, e.g., lidar and radar data.

In this paper, sensor data from lidar, radar, stereo camera, and thermal camera are fused with a probabilistic 2D occupancy grid map. This data representation has been chosen as its non-parametric property allows the representation of diffuse agricultural environments. Furthermore, it simplifies path planning and is already a standard in the automotive industrial research (Garcia et al., 2008; Bouzouraa and Hofmann, 2010; Konrad et al., 2012; Winner, 2015). Traditionally, occupancy grid maps represent traversable and non-tranversable areas in a binary decision. The occupancy grid mapping used in this paper, however, is applied in a much richer fashion, due to the extension to multiple semantical layers. Thus, techniques for finding an optimal path, such as the A* search algorithm, cannot be directly applied. Furthermore, the finding of an optimal path online in agricultural processes is not mandatory, due to the fact that a full area coverage is aimed, which is inherently defined by the topology and shape of the field. The quantification of the area which lies ahead, and, therefore, the prediction of process characteristics is of higher interest. While the direct deduction from the semantical grid maps becomes unfeasible, a so-called decoding for inferring process-relevant information is introduced.

In this work, generative models for inferring process-relevant information out of the mapped sensors’ detections are used. Generative models have a number of applications in prediction, missing data imputation or probabilistic inference (Rabiner, 1989; Hinton and Salakhutdinov, 2006). One mathematical framework of generative models is the Hidden Markov Model (HMM) which is able to respect the time-domain and noisy sensor data of a process. Applications to robotics and grid maps have shown the incorporation of learning and decoding of hidden property information from the environment which makes HMMs a suitable approach to infer properties out of the semantical grid maps (Stachniss, 2009; Walter et al., 2013; Vasquez et al., 2017).

## 3 Method

In the following, each step from the system architecture in Figure 1 is explained in detail. Section 3.1 describes the recording setup including sensor specifications. Section 3.2 describes the fusion and mapping approach that takes in inverse sensor models and combines these to generate fused obstacle maps. Section 3.3 describes the inverse sensor models, consisting of sensor-specific detection algorithms and transformations to 2D occupancy grid maps. Finally, section 3.4 describes the process evaluation that uses the fused maps to decode process-relevant properties along the trajectories of the tractor.

### 3.1 Sensor Platform

**Figure 2:**
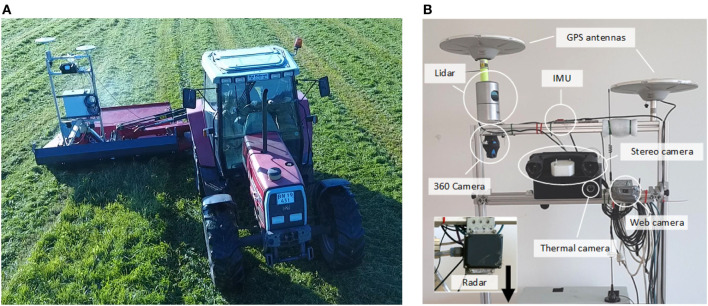
Recording platform. **(A)** Platform attached to tractor-mounted mower. **(B)** Sensor setup. Reprinted from Kragh et al. (2017) with permission.

The sensor suite presented by Kragh et al. (2017) was used to record multi-modal sensor data. The dataset has recently been made publicly available.^2^ It includes lidar, radar, stereo camera, thermal camera, IMU, and GNSS.^3^ The sensors were fixed to a common platform and interfaced to the Robot Operating System (ROS) (Koubaa, 2016). A tractor-mounted setup and a close-up of the platform are shown in Figure 2.

The exteroceptive sensors and their properties are listed in Table 1. Proprioceptive sensors used for localization included a Vectornav VN-100 IMU and a Trimble BD982 dual antenna GNSS system. All sensors were synchronized in ROS. Lidar, stereo camera, and thermal camera were registered before recording in a semi-automatic calibration procedure (Christiansen et al., 2017). All remaining sensors were registered by hand, by estimating extrinsic parameters of their positions. Global localization from IMU and GNSS was obtained with the robot_localization package (Moore and Stouch, 2014) available in ROS, by simply concatenating the world referenced position and orientation. The overall localization accuracy was thus determined by the sensor accuracies of the GNSS (8 and 15 mm SDs for horizontal and vertical positions, and <0.5° for yaw) and IMU (1.0° SDs for roll and pitch).

**Table 1:** Sensors.

Sensor	Model	Resolution	FOV (°)	Range (m)	Data rate (fps)
Stereo camera	Multisense S21, CMV2000	1,024 × 544	85 × 50	1.5 – 50	10
Web camera	Logitech HD Pro C920	1,920 × 1,080	70 × 43	n/a	20
360° camera	Giroptic 360cam	2,048 × 833	360 × 292	n/a	30
Thermal camera	Flir A65, 13 mm lens	640 × 512	45 × 37	n/a	30
Lidar	Velodyne HDL-32E	2,172 × 32	360 × 40	1–100	10
Radar	Delphi ESR	32 targets/frame	90 × 4.2	0–60	20
			20 × 4.2	0–174	

*Adapted from Kragh et al. (2017) with permission*.

### 3.2 Fusion and Mapping

Occupancy grid maps are used in static obstacle detection for robotic systems, which is a well-known and a commonly studied scientific field (Hähnel, 2004; Thrun et al., 2005; Stachniss, 2009). They are components of almost all navigation and collision avoidance systems designed to maneuver through cluttered environments. Another important application is the creation of obstacle maps for traversing unknown areas and the recognition of known obstacles, thereby supporting localization. Recently, occupancy grid maps have been applied to combine lidar and radar in automotive applications with the goal of creating a harmonious, consistent, and complete representation of the vehicle’s environment as a basis for advanced driver assistance systems (Garcia et al., 2008; Bouzouraa and Hofmann, 2010; Winner, 2015).

#### 3.2.1 Occupancy Grid Mapping

Two-dimensional occupancy grid maps (OGM) were originally introduced by Elfes (1990). In this representation, the environment is subdivided into a regular array or a grid of quadratic cells. The resolution of the environment representation directly depends on the size of the cells. In addition to this compartmentalization of space, a probabilistic measure of occupancy is associated with each cell. This measure takes any real number in the interval [0,1] and describes one of the two possible cell states: unoccupied or occupied. An occupancy probability of 0 represents a space that is definitely unoccupied, and a probability of 1 represents a space that is definitely occupied. A value of 0.5 refers to an unknown state of occupancy.

An occupancy grid is an efficient approach for representing uncertainty, combining multiple sensor measurements at the decision level, and for incorporating different sensor models (Winner, 2015). To learn an occupancy grid *M* given sensor information *z*, different update rules exist (Hähnel, 2004). For the authors’ approach, a Bayesian update rule is applied to every cell *m*∈*M* at position (*w*,*h*) as follows: Given the position *x_t_* of a vehicle at time *t*, let *x*_1:_*_t_* = *x*_1_, …, *x_t_* be the positions of the vehicle’s individual steps until *t*, and *z*_1:_*_t_* = *z*_1_, …, *z_t_* the environmental perceptions. For each cell *m* of the occupancy probability grid *P*(*m*|*z*_1:_*_t_*,*x*_1:_*_t_*) represents the posterior probability that this cell is occupied by an obstacle. Thus, occupancy probability grids seek to estimate

(1) $$P\left(m|z_{1:T},x_{1:T}\right)=\text{Od}{\text{d}}^{-1}\left(\overset{T}{\underset{t=1}{\prod }}\text{ Odd}\left(P\left(m|z_{t},x_{t}\right)\right)\right),\text{Odd}\left(P\left(m|z_{t},x_{t}\right)\right)=\frac{P\left(m|z_{t},x_{t}\right)}{1-P\left(m|z_{t},x_{t}\right)}.$$

This equation already describes the online capable, recursive update rule that populates the current measurement *z_t_* to the grid, where *P*(*m*|*z*_1:_*_t_*, *x*_1:_*_t_*) is the so-called inverse sensor model (ISM). The ISM is used to update the OGM in a Bayesian framework, which deduces the occupancy probability of a cell, given the sensor information.

#### 3.2.2 Extension to Agricultural Applications

Contrary to robotic or automotive applications, OGM techniques are not directly applicable to agricultural applications. Common applications want to detect non-traversable areas or objects occupying their paths. Such unambiguous information is used to quantify the whole environment sufficiently for all derivable tasks such as path planning and obstacle avoidance. When assumptions like a flat operational plane or minimum obstacle heights are made, the projection of the sensor’s frustum to the ground plane is sufficient for all tasks.

In agricultural applications, a crucial task is to quantify the environment as the machines act on and process it. This involves features such as processed areas, processability, crop quality, density, and maturity level in addition to traversability. In order to map these features, single occupancy grid maps are no longer sufficient. Instead, semantical occupancy grid maps (SOGM) that allow different classification results to be mapped are used. Furthermore, sensor frustums are no longer oriented parallel to the ground, but rather oriented at a downward angle to gather necessary crop information (Korthals et al., 2017b).

**Figure 3:**
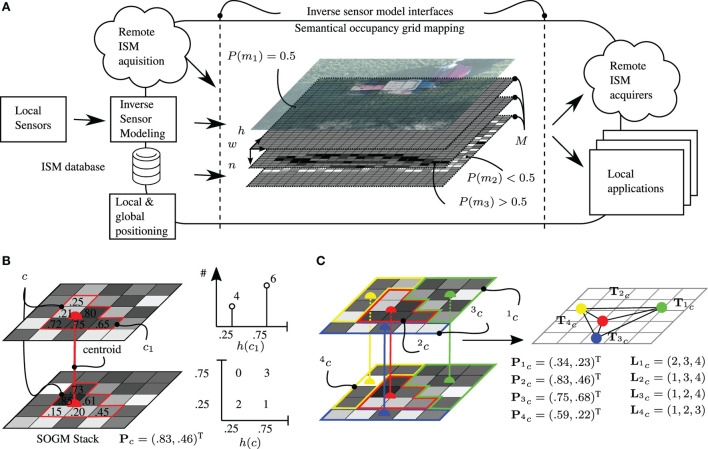
Semantical OGM framework and supercell clustering. **(A)** Semantical occupancy grid mapping framework. **(B)** Supercell with *N* = 2 layers and corresponding histograms with *K* = 2 bins. **(C)** Conversion of supercells to a graph of centroids labeled with feature vectors.

The extension to SOGM or inference grids is straightforward and defined by an OGM *M* with *W* cells in width, *H* cells in height, and *N* semantical layers (see Figure 3A):

(2) $$M:\left\{1,\ldots ,W\right\}\times \left\{1,\ldots ,H\right\}\to m={\left[0,1\right]}^{N}.$$

Compared to a single layer OGM which allows the classification into three states {occupied, unoccupied, unknown}, the SOGM supports a maximum of 3 *^N^* different states allowing much higher differentiability in environment and object recognition. The corresponding ISMs are fused by means of the occupancy grid map algorithm to their *n*th associated semantical occupancy grid.

The location of information in the maps is required to be completed by *mapping under known poses* approaches (Thrun et al., 2005). The ISMs are mapped locally in the maps while the maps themselves are globally referenced enabling consistent storing and loading of information. Furthermore, it allows smooth local mapping in the short term without discrete jumps caused by global positioning systems using a Global Navigation Satellite System (GNSS) (Korthals et al., 2017b).

#### 3.2.3 Mapping Capabilities

SOGMs contain a generic representation of the environment. However, for many applications, only part of this vast amount of information is required. Therefore, in the following, we introduce three methods of fusing SOGMs. The first two methods are cell-wise layer fusions given in equations (3) and (4), while the third method is a cell-clustering technique working across layers given in equation (5). These are used in the evaluation for binary traversability assessment, class-specific obstacle mapping, and process evaluation.

The first approach introduced in equation (3) is based on a super Bayesian independent opinion pooling *P*_B_ (Pathak et al., 2007). It is applicable for the case when separate SOGMs with identical feature representations (same object classes) are maintained. Second, equation (4) introduces a non-Bayesian maximum pooling fusion method *P*_M_ is applied to heterogeneous feature representations (varying object classes) (Liggins et al., 2001). The fusion techniques are cell-wise and, therefore, do not introduce any clustering:

(3) $$P_{\text{B}}\left(m\right)=\frac{1}{1+\prod_{n}\;\frac{1-P\left(m_{n}\right)}{P\left(m_{n}\right)}},$$

(4) $$P_{\text{M}}\left(m\right)=\underset{n}{\text{max}}\;P\left(m_{n}\right).$$

Unlike single-layer OGM approaches, an SOGM incorporates multiple OGMs with varying classes residing in the map storage. For many applications cell-wise consideration, which is the disregarding of the cells’ surroundings, is not a feasible approach due to noisy or sparse data and potential positional offsets between layers. Thus, clustering on SOGMs was introduced by Korthals et al. (2017a) using a Supercell Extracted Variance Driven Sampling (SEVDS) algorithm, which tends to find clusters that consist of mainly non-contradicting cells:

(5) $$H\left(c\right)=D\left(c\right)+\Gamma G\left(c\right)\text{ with }D\left(c\right)=\sum_{n=1}^{N}e_{n}\left(\mathrm{var}\left(h\left(c\right)\right)\right).$$

In equation (5), *c* is the supercell of interest and *G* is the contour function, which can be smoothed via the scalar factor Γ. The distribution term *D* of a supercell *c* is defined as the sum of Eigenvalues *e* of the covariance matrix of the probability histogram *h*(*c*) (see Figures 3B,C). The contour term *G* is taken from Van den Bergh et al. (2015) and evaluates cell-wise updates that penalize irregular shapes, e.g., a single cell extending into an adjacent supercell. A scalar factor of Γ = 1 is used as in the original paper.

As depicted in Figure 3C, for every found supercell, a triple C = (**T***_c_*,**L***_c_*,**P***_c_*) consists of its centroid location **T***_c_*, a list of adjacent supercells **L***_c_*, and a feature vector ${\text{P}}_{c}\in R^{N}$, with *N* being the number of SOGM layers. Odd(**P***_c_*) is calculated as:

(6) $$\text{Odd}\left({\text{P}}_{c}\right)={\left(\underset{m\in c_{1}}{\prod }\text{ Odd}\left(P\left(m\right)\right),\ldots ,\underset{m\in c_{N}}{\prod }\text{ Odd}\left(P\left(m\right)\right)\right)}^{\text{T}}.$$

#### 3.2.4 Recency Weighting for Dynamic Obstacles

When evaluating the detection dynamic obstacles, static obstacle detections are ignored by introducing recency weighting to the mapserver via two new parameters. A *ForgetValue* indicates the amount of temporal memory in the map. A value of 0 indicates no forgetting, such that all information remains in the map, once it is introduced. A value of 1, however, indicates total forgetting (no memory), such that the map is cleared every time the forgetting is applied. The second parameter is a *ForgetRate* that indicates the rate at which the forgetting is applied. A rate of 2 means that two times every second, all cells in the map are updated with respect to the *ForgetValue*:

(7) $$P\left(m_{t}\right)=\left(P\left(m_{t^{-}}\right)-0.5\right)\times \left(1-\mathrm{ForgetValue}\right)\underset{n\in N}{\sum }\;\gamma \left(t-\frac{n}{\mathrm{ForgetRate}}\right)+0.5.$$

First, $P\left(m_{t^{-}}\right)$ is centralized at 0 where *t*^–^ addresses the cell property just before the update. γ indicates the discrete Dirac function which builds up the sampling function with its sampling rate *ForgetRate*. With every forgetting step, the updated posterior probability converges to 0.5 which indicates no knowledge over the cell *m*. Thus, equation (7) is a basic exponential smoothing filter with $P\left(m_{t^{-}}\right)$ being the start excitation (Biber, 2005).

### 3.3 Inverse Sensor Models

In the following, individual inverse sensor models (ISM) are introduced and explained in detail for each of the sensors. An ISM consists of an algorithm for detecting a number of object categories and a mapping to align detection information using a 2D occupancy grid map (OGM) in the local sensor frame.

#### 3.3.1 Cameras

In this section, multiple ISMs are described for the stereo camera and thermal camera. First, the individual detection algorithms operating on image data are explained. Then, two procedures for aligning detections to OGMs are proposed.

##### 3.3.1.1 Detection Algorithms

**Figure 4:**
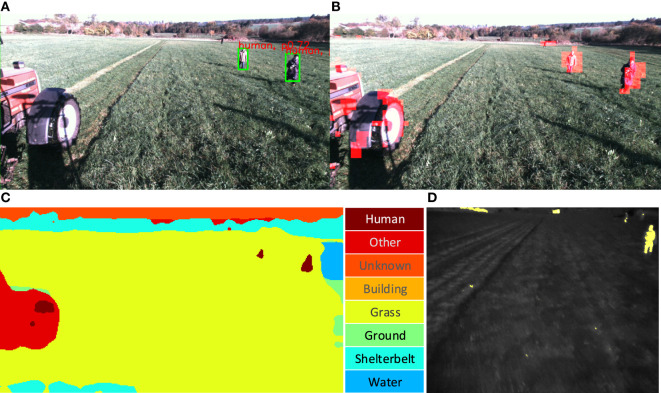
Camera detections for stereo and thermal camera. Written and informed consent was obtained from all depicted individuals. **(A)** Object detection using YOLO. **(B)** Anomaly detections (highlighted with red) using DeepAnomaly. **(C)** Semantic segmentation using FCN. **(D)** Thermal camera detections (highlighted with yellow) using HeatDetection.

A total of four detection algorithms for the stereo camera have been used; Locally Decorrelated Channel Features (LDCF) for pedestrian detection (Dollár et al., 2014), an improved version of You Only Look Once (YOLO) (Farhadi and Redmon, 2017; Redmon et al., 2016) for object detection, a Fully Convolutional Neural Network (FCN) for semantic segmentation (Long et al., 2015), and DeepAnomaly (Christiansen et al., 2016a) for anomaly detection. The algorithms all use a single color image from the stereo camera. For the thermal camera, a heat detection algorithm (HeatDetection) is used to detect objects that are warm compared to the background using a dynamically adjusted threshold (Christiansen et al., 2014). Figure 4 presents examples of output predictions from the detection algorithms.

LDCF is a pedestrian detection algorithm delimiting instances by bounding boxes with fixed aspect ratios. The model is trained on the INRIA Person Dataset (Dalal and Triggs, 2005). The detector is publicly available in a MATLAB-based framework by Dollar (2015) and has been converted to C + + and wrapped in a ROS-package^4^ (Kragh et al., 2016).

YOLO is a deep learning-based object detector delimiting instances by bounding boxes of variable aspect ratios. The detector is developed in the deep learning framework Darknet (Redmon, 2013) and trained on ImageNet (Russakovsky et al., 2015) and Microsoft COCO (Lin et al., 2014) for detecting 80 object categories. For running the algorithm within the proposed framework, a ROS-package^5^ has been developed which also applies a remapping of the 80 object classes into three classes (human, object, and unknown).

FCN uses the backbone of VGG (Simonyan and Zisserman, 2014) to make a fully convolutional semantic segmentation algorithm that classifies all pixels in an image. The model is developed in Caffe (Jia et al., 2014) and is publicly available.^6^ The model is trained on the 59 most frequent classes of the Pascal Context dataset (Mottaghi et al., 2014). Unlike the more popular Pascal VOC dataset (Everingham et al., 2013) with only 20 object classes, Pascal Context provides full image annotations of 407 classes. In Christiansen et al. (2016b), the 59 object classes are remapped to only 11 classes to investigate semantic segmentation in an agricultural context. In Kragh et al. (2016), the detector has been wrapped in a ROS-package.^7^ In the current work, predictions are remapped to six classes (human, object, grass, ground, vegetation, and undefined).

DeepAnomaly is a deep learning-based detection algorithm for detecting anomalies (Christiansen et al., 2016a). The backbone is AlexNet (Krizhevsky et al., 2012) trained on ImageNet, and the anomaly detector is modeled using 150 images from the dataset in Christiansen et al. (2017). The output consists of coarse predictions of the whole image.

HeatDetection uses a heat detection principle from Christiansen et al. (2014) for detecting warm objects using a thermal camera. The median temperature is determined for all image pixels of the current image, and the dynamic threshold is defined 3.0°C above the median temperature. In this work, the median temperature is determined for the bottom 80% of the image to not include image sections of the sky. Subtracting the image by the dynamic threshold and clipping values below zero results in a heat map of how much each pixel has exceeded the dynamic threshold. A ROS-package is publicly available.^8^

##### 3.3.1.2 Mapping of Detections to OGM

**Figure 5:**
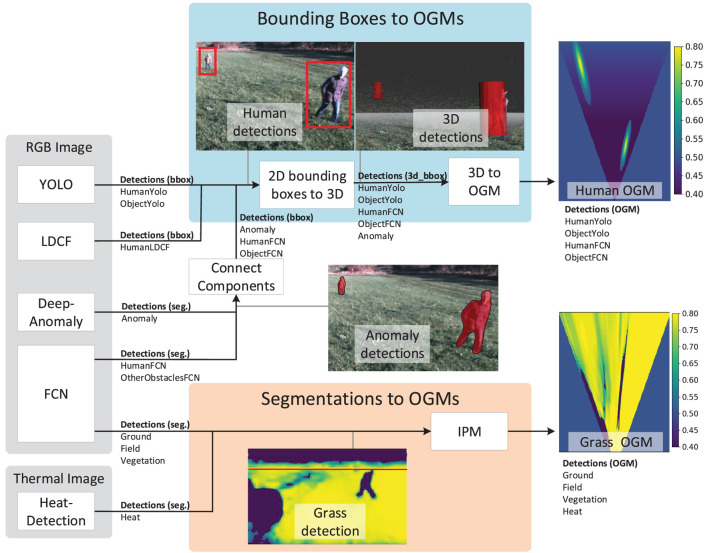
Converting detections to OGMs. Written and informed consent was obtained from all depicted individuals.

Camera detections are mapped to an OGM representation (Korthals et al., 2017b) using two procedures as presented in Figure 5. The top branch denoted *Bounding Boxes to OGMs* is for mapping detections represented by bounding boxes. The bottom branch denoted *Segmentations to OGMs* is for mapping segmented image detections. Finally, a few exceptions exist for DeepAnomaly and two FCN classes where segmented elements are converted to bounding box representations using a connected component module before mapping to OGM. The code has been made publicly available as ROS packages.^9^,^10^ Below, the two branches are described in more detail.

###### 3.3.1.2.1 Bounding Boxes to OGMs

This procedure maps detections to OGMs by first converting 2D bounding boxes to 3D cylinders. First, the distance to an object is estimated using depth from stereo matching. The distance is defined as the median depth inside the bounding box. The estimated distance is assigned to each bounding box corner and mapped to 3D using conventional camera geometry. Bounding box corners are converted to a cylinder represented by a center position, width, and height. Finally, 3D detections are mapped to an OGM as the output of the top branch in Figure 5.

Various heuristics are used for modeling the OGM’s uncertainties. Areas outside the camera’s field of view (FOV) are set to 0.5. Areas inside the FOV with no detections w.r.t. *m* are set to 0.4 indicating lower probabilities of occupancy. Detections w.r.t. *m* are given a value between 0.5 and 0.8 to indicate that the areas are occupied by the corresponding detections. A value of 0.5 represents the minimum prediction or class probability by a detection algorithm, whereas a value of 0.8 represents the maximum. Values in between are scaled linearly. A maximum value of 0.8 was chosen to avoid early saturation under fusion.

Imprecise localization of a detection is modeled by a Gaussian distribution. For a camera, the uncertainty of distance (radial coordinate) and angle (angular coordinate) to the object are independent. This is incorporated by modeling each polar coordinate (radial and angular) with independent uncertainties. In Figure 5, the localization uncertainty caused by the radial coordinate is larger than the uncertainty caused by the angular coordinate.

A detection algorithm is less likely to detect distant obstacles or to guarantee that an obstacle is not there. To model this, the certainty of not detecting an obstacle is reduced linearly by the distance from the nearest to the most distant grid cells. In Figure 5, the probability increases linearly with distance from 0.4 to 0.5.

###### 3.3.1.2.2 Segmentations to OGMs

Inverse perspective mapping (IPM) is used for mapping image segmentations to a grid map. IPM projects an image from the camera frame to the ground plane surface using a geometrical transformation (Bertozzi and Broggi, 1998; Konrad et al., 2012). The purpose of IPM is to remove/inverse the perspective effect by changing the viewpoint from the camera to a bird’s-eye view. Areas outside the camera FOV are set to 0.5. Areas inside the FOV with no detections are set to 0.4. Detections are given a value between 0.5 and 0.8 to indicate that the areas are occupied.

The IPM algorithm is able to approximate the actual mapping for flat elements on the surface such as grass. However, elements protruding or positioned above the ground surface (e.g., humans and many obstacles) are imprecisely mapped. For this reason, segmentations of anomalies, humans, and other obstacles are converted to bounding boxes using a connected component module as illustrated in Figure 5. The OGM for a grass-segmented image is presented in the bottom of the figure.

#### 3.3.2 Lidar

The inverse sensor model for the lidar sensor consists of a detection algorithm and a mapping to align detection information to a local 2D occupancy grid map (OGM) in the sensor frame. The detection algorithm operates directly on 3D point clouds with approximately 70,000 points/frame generated at 10 fps by the Velodyne HDL-32E lidar. First, 13 features are calculated per point using neighborhood statistics that depend on local point densities (Kragh et al., 2015). Second, a Support Vector Machine (SVM) classifies each point as either *ground*, *vegetation*, or *object*. It further assigns probability estimates (Wu et al., 2004) to each class to describe the certainty of each classification. The SVM classifier was trained on the same data used in Kragh et al. (2015).

The mapping from detection probabilities to a local 2D grid is handled by projecting and resampling 3D points into 2D grid cells. For each 2D grid cell, class probabilities of all 3D points whose flattened projection lies inside are averaged and normalized such that the three class probabilities sum to 1. This results in three 2D probability grids: $P_{\mathrm{object}}^{*}$, $P_{\mathrm{vegetation}}^{*}$, and $P_{\mathrm{ground}}^{*}$. The three classes are combined into two OGMs (lidar-SVM-object and lidar-SVM-vegetation) by incorporating the *ground* probabilities into the *object* and *vegetation* classes probabilistically with Bayesian fusion. For each grid cell *m* in an OGM, the log odds ratio of, e.g., the *object* class is:

(8) $$\begin{array}{lll} \text{log Odd}\left(P_{\mathrm{object}}\left(m\right)\right)=\text{log Odd}\left(P_{\mathrm{object}}^{*}\left(m\right)\right)+\text{log Odd}\left(1-P_{\mathrm{ground}}^{*}\left(m\right)\right) & & \\ \;\;\;\;=\text{log}\left(P_{\mathrm{object}}^{*}\left(m\right)\right)-\text{log}\left(1-P_{\mathrm{object}}^{*}\left(m\right)\right)\; & & \;\\ \;\;\;\;\;-\text{log}\left(P_{\mathrm{ground}}^{*}\left(m\right)\right)+\text{log}\left(1-P_{\mathrm{ground}}^{*}\left(m\right)\right). \end{array}$$

**Figure 6:**
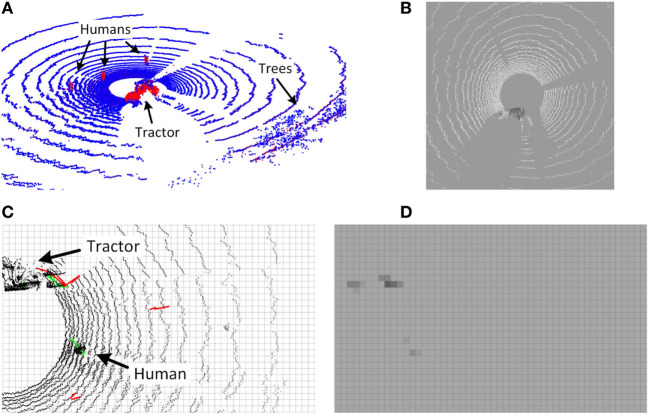
Lidar and radar detections and OGMs. **(A)** Point cloud with pseudo-colored probability estimates of the *object* class. Blue and red denote low and high probabilities, respectively. **(B)** Resulting lidar OGM for the *object* class illustrating low (bright) and high (dark) probabilities. **(C)** Radar detection example with confirmed (green) and unconfirmed (red) radar tracks overlaid on point cloud. **(D)** Resulting radar OGM.

Figure 6A shows an example of a point cloud colored by *object* probabilities from the SVM classifier, while Figure 6B shows the corresponding *object* OGM.

#### 3.3.3 Radar

The Delphi ESR automotive radar provides a list of up to 32 targets for each frame. Each target is represented by an angle, a range, and an amplitude. Most targets, however, represent internal noise in the radar and have low amplitudes. Simply filtering out these targets with a threshold eliminates radar returns from low-reflective objects such as humans and animals. Therefore, instead the approach from the authors’ previous paper (Kragh et al., 2016) was used in combination with a tracking algorithm between subsequent frames known as the Kuhn–Munkres assignment algorithm (Munkres, 1957). Only radar targets that are less than 2 m apart between two consecutive frames are associated. A track *i* is described by its current position and its track length *L_i_*. It is confirmed when *L_i_* > *L*_min_ = 3 m and converted to a detection pseudo-probability by:

(9) $$P_{\mathrm{radar},i}=0.5+0.5\frac{L_{i}-L_{\text{min}}}{L_{i}}.$$

The addition of 0.5 makes the detector report only positive information of occupancy, thus not indicating absence of objects. The mapping from detection probabilities to a local 2D grid is handled by converting from polar to Cartesian coordinates and resampling into 2D grid cells. For each 2D grid cell, class probabilities of all detections lying inside are averaged. This results in a 2D probability grid $P_{\mathrm{radar}}^{*}$. Finally, the log odds ratio for each grid cell *m* in the radar OGM (radar-tracking) can be expressed as:

(10) $$\text{log Odd}\left(P_{\mathrm{radar}}\left(m\right)\right)=\text{log}\left(P_{\mathrm{radar}}^{*}\left(m\right)\right)-\text{log}\left(1-P_{\mathrm{radar}}^{*}\left(m\right)\right).$$

Figure 6C shows an example of confirmed (green) and unconfirmed (red) radar tracks overlaid on the corresponding point cloud, while Figure 6D shows the resulting radar OGM.

### 3.4 Process Evaluation

Farming scenarios are commonly well-defined and the trajectories are always planned in advance to yield optimal efficiency. However, the field may consist of many different properties that can only be revealed by sensing the current environment. Common properties are *cropable*, *traversable*, or *non-traversable*, where of course the yield itself is of special interest.

The environment of the field is made up of structures in space that are sensed by diverse sensors. While the well-defined vehicle trajectory traverses this area, this path is of particular interest to forecast implement parameters or steering suggestions. Furthermore, due to imperfections in sensor calibration, registration, and synchronization, areas of detections may not always overlap and will, therefore, always have spots where only certain sensors sense a property. This phenomenon evolves along the frustum and, therefore, along the planned trajectory. Thus, changes in real-world scenes are sequential in space, and the sequential nature can be used to learn property relationships between the various semantical occupancy grid map (SOGM) layers to analyze scenes. In this section, a hierarchical model that maps an observed SOGM along a trajectory to properties is presented.

**Figure 7:**
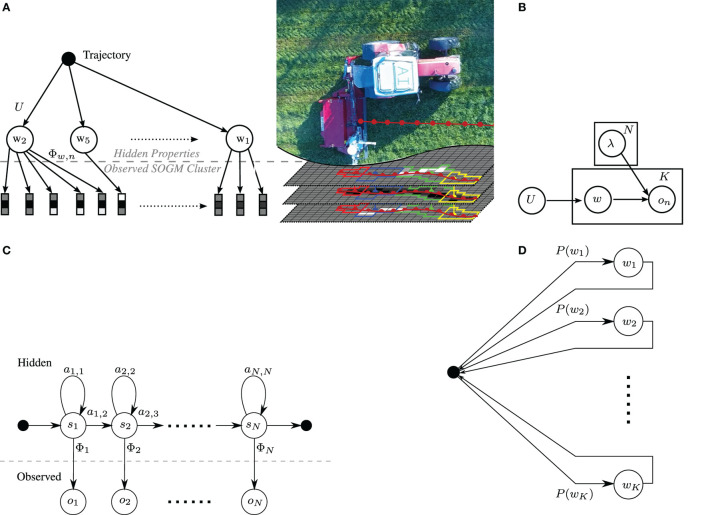
Generative model and Hidden Markov model framework for identifying properties in the mapped data. **(A)** Conceptual representation of the proposed framework with the generative sampling on the left and a corresponding scenario with observations along the red tractor trajectory on the right. **(B)** Generative model for property identification. Only *o_n_* is observed. **(C)** Left–right structure of one intra-property model λ_*w*_ for the inter-property model (exit transition per state is not visualized). **(D)** Ergodic inter-property model of the HMM.

Figure 7A shows the kind of structured information that is envisioned parsing from the trajectory over an SOGM. The lowest level corresponds to the feature vectors extracted from equation (6). The middle layer corresponds to a property (e.g., *cropable*), and the top root node represents the trajectory. The cost of obtaining such hierarchical annotations would be very high due to the complexity of the annotation task. Typically, agricultural datasets are not labeled with all desired properties. As a result, models for learning such structures should also be able to operate in an unsupervised framework.

The problems to address are twofold. *Learning*: in order to categorize or classify mappings along the trajectory into properties, statistical characterizations of the patterns of observation sequences must be learned. *Classification*: given observations along a trajectory, an algorithm is needed to classify these into properties.

#### 3.4.1 A Generative Model for Inducing Properties Over SOGMs

For the given task of path traversal, a hierarchical approach is targeted that not only models the single property at a certain location, but also the whole object itself. The probability making observation O = (*o*_1_,…,*o_IJ_*) with property *w* can be expressed as the joint probability

(11) $$P\left(O,w;\lambda \right)=\prod_{i=1}^{I}\;P\left(w_{i}\right)\prod_{j=1}^{J}\;P\left(o_{j}|w_{i};\lambda \right)$$

with the hidden variable *w*, *P*(*w*) being the discrete property probability, and λ being the generative property model for the observed feature vector O. The amount of properties along a path are enumerated by *I* while the length of a single property is denoted by *J*.

The inter-property model $\lambda_{w}=\left(S,O,A,\Phi ,\Pi \right)$ is a corresponding Hidden Markov Model (HMM) with states *s*, observations O, transition probability *A*, emission probability Φ, and start probability Π for every single property *w*. The emission probability is modeled as a beta mixture model (BMM) over the *N* semantical occupancy grids with *δ* as normalization weight and the beta function B with its parameters ***α*** and ***β***:

(12) $$\Phi \left(\delta ,\alpha ,\beta \right)=\sum_{n=1}^{N}\;\delta_{n}B\left(\alpha_{n},\beta_{n}\right).$$

At the lowest level of the hierarchical structure specified by the model in Figure 7A is a sequence of probabilistic feature vectors. In reality, there are infinitely many feature vectors. Moreover, due to imperfections in localization and mapping, regions among semantical layers may not overlap perfectly and can be noisy as depicted in Figure 7A.

As discussed before, it is expected that trajectories are composed of a sequence of semantically meaningful properties that manifest themselves in various property-compositions. The feature vectors themselves can be directly modeled as a BMM as stated in equation (12). While a direct classification might be suitable, a sequence along the trajectory (which is along the field of view) may represent a true underlying property even better and can only be revealed when taking spatially earlier readings into account. Therefore, a generative model is introduced in Figure 7B, where the interpretable properties generate probability feature vectors (the features of a supercell).

The distribution of properties in the field will be stochastic in nature (e.g., a trajectory may contain segments of crop, weed, and non-traversability), and the distribution of the feature vectors themselves is beta-distributed and property-dependent. While the number of such properties is expected to be very large, it is assumed that for a given dataset a limited number of properties can describe the property space fairly well.

The generative model is shown in Figure 7B. *K* properties in the vocabulary and feature vectors ${\text{P}}_{c}\in R^{N}$ are assumed (see equation (6)). A set of *T* trajectories can be generated as follows: for each trajectory *t*, *I* properties are drawn from a unigram distribution *U*. We then draw *J* feature-vectors from the specific generative property-model. Thus, in this model, each trajectory is a bag of properties and each occurrence of a property is a sequence of feature-vectors. The resulting hirachical model is shown as a concatenation of Figure 7D, as an ergodic model for the inter-properties, and Figure 7C, as left-right model for the inter-property realization.

#### 3.4.2 Model Estimation and Decoding

An HMM, as shown in Figure 7C, for each of the *K* properties is produced. It is modeled as a left-right structure with an additional exit transition for each state to follow the aforementioned idea of non-perfectly overlapping detections. Thus, property burn-in, settling, and burn-out behaviors can be modeled in the beginning, middle, and end of the trajectory. Therefore, a minimum of three states *s* are necessary to model these behaviors for every property *w*. Since properties may have very diverse features in the start and end sequence, all states have their own emission probability.

The HMMs for the properties are now put together as shown in Figure 7D. For the sake of simplicity, a black circle represents the hub for all property transitions in the ergodic model. *P*(*w_k_*) represents the probability of the property *w_k_*. This approach is trained in a supervised fashion and thus, the objective function for one property *w* tends to find the most likely model λ_*w*_, given an observation O and its corresponding (GT) sequence S

(13) $$\lambda_{w}={\text{argmax}}_{\lambda_{w}}P\left(O,S|\lambda_{w}\right).$$

Equation (13) can be estimated by *instance counting*, which counts the hidden state transitions and output states, and uses the relative frequencies as estimates for the transition probabilities of λ_*w*_. The inter-property model can be trained in the same way. Given the GT, the parameters α and β can be directly determined by the *Method of Moments*. For decoding, the likelihood $P\left(O|\lambda_{w}\right)$ that a given model λ_*w*_ has produced a given observation sequence O is calculated by the Viterbi algorithm (Rabiner, 1989).

## 4 Evaluation

In this section, we evaluate the proposed architecture for obstacle detection, recognition, and mapping on static and dynamic obstacles, individually. Furthermore, we evaluate the process evaluation on the mapped data with a spatial resolution of 10 cm per cell.

### 4.1 Dataset

**Figure 8:**
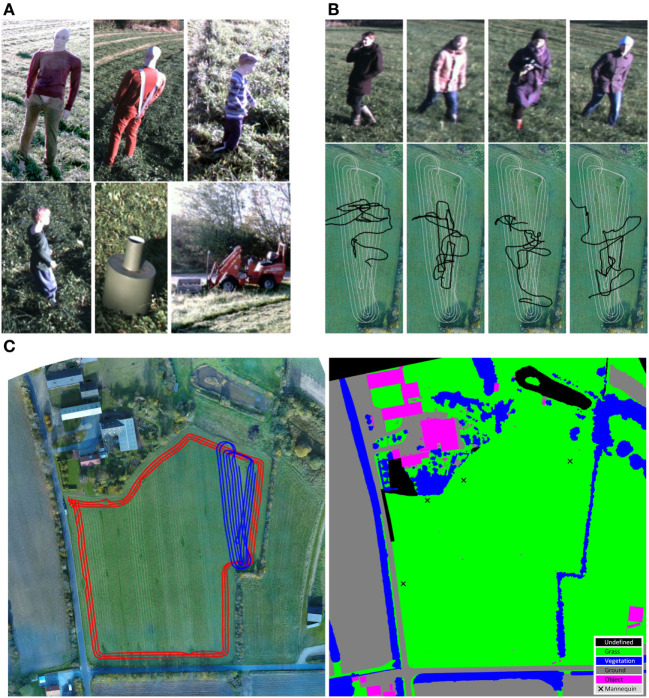
FieldSAFE dataset. **(A)** Examples of static obstacles. **(B)** Examples of moving obstacles (from the stereo camera) and their paths (black) overlaid on tractor path (gray). **(C)** Colored and labeled orthophotos. Left: orthophoto with tractor tracks overlaid. The red track includes only static obstacles, whereas the blue track also has moving obstacles. Right: annotated orthophoto with pixel-wise labels. Adapted from Kragh et al. (2017) with permission. Written and informed consent was obtained from all depicted individuals.

The publicly available FieldSAFE dataset (Kragh et al., 2017) for multi-modal obstacle detection in agricultural fields was used for the evaluation. The dataset includes 2 h of recording during mowing of a grass field in Denmark. Figure 8A illustrates examples of static obstacles in the dataset, whereas Figure 8B shows examples of dynamic obstacles (humans) and their GT traversed paths overlaid on the path of the tractor. Figure 8C shows a static orthophoto of the field together with pixel-wise manually labeled GT classes. In the following section, the annotated orthophoto is used as ground truth for evaluating the proposed architecture.

### 4.2 Static Scenario

Two different evaluations have been performed: evaluation **A** for detecting process-relevant classes exclusively, and evaluation **B** for detecting occupied areas with respect to traversability.

For evaluation **A**, GT labels were grouped into four different process-relevant classes (*Vulnerable obstacles*, *Processable*, *Traversable*, and *Non-traversable*). The *Vulnerable obstacles* class included GT label *Mannequin* and covered regions with which a collision must be avoided under any circumstance. The *Processable* class included GT label *Grass* and represented the crop. The *Traversable* class included GT labels *Grass* and *Ground* and represented areas that could be traversed by the vehicle. Finally, the *Non-traversable* class included GT label *Vegetation* and represented areas that must be avoided to not damage the vehicle. For evaluating the process-relevant detection, each of the four classes was considered in its own property map. Included GT classes were marked as *occupied*, whereas all other classes were treated as *unknown*.

For evaluation **B**, GT labels were grouped into three different properties (*occupied*, *unoccupied*, and *unknown*) according to their traversability. The labels *Vegetation*, *Mannequin*, and *Object* were combined to the *occupied* property. The label *Undefined* was considered an *unknown* property, whereas the remaining classes *Ground* and *Grass* were combined to the *unoccupied* property.

**Figure 9:**
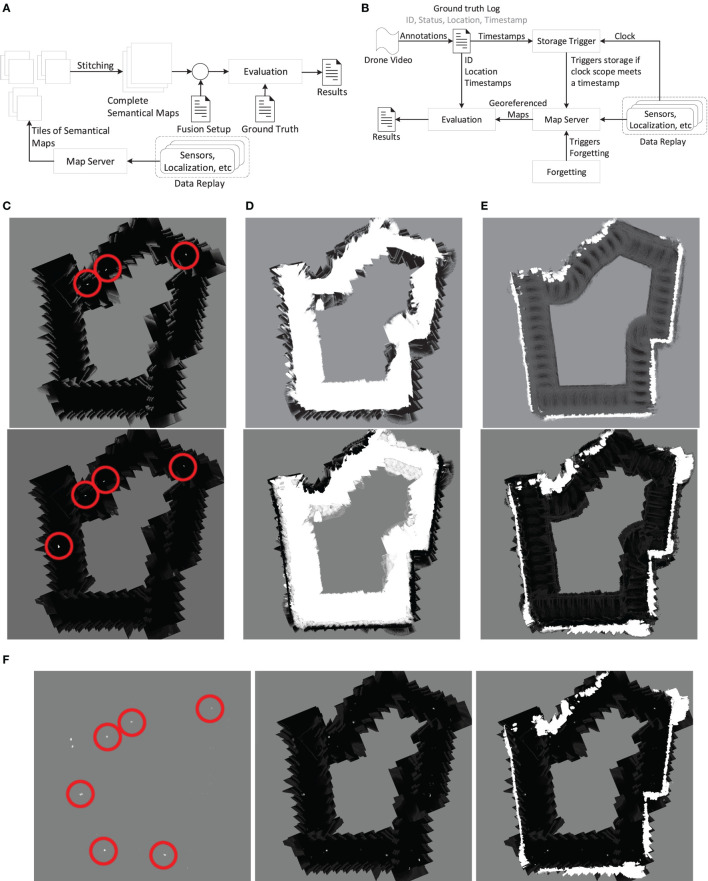
Examples for different stitched mapping results for different evaluations of Table 2**A (C–E)**, Table 2**B (F)**, and evaluation pipelines **(A,B)**. Red circles emphasize correct object/mannequin detections. Grayscale encoding: black ≙ *occupied*, white ≙ *unoccupied*, gray ≙ *unknown*. **(A)** Evaluation pipeline from static recording to evaluation with stitching. **(B)** Evaluation pipeline from dynamic recording using drone video and recorded data as input. **(C)** cam-YOLO-human (top) and fused human class (bot.). **(D)** cam-FCN-ground (top) and fused ground class (bot.). **(E)** lidar-SVM-veg. (top) and fused vegetation class (bot.). **(F)** radar-tracking (left), Bayesian fusion among class (mid.), and complete fused map (right).

To quantify the detection of static obstacles and to compare it against the GT data from subsection 4.1, the evaluation pipeline from Figure 9A was applied. The mapserver’s maps, which contain all fused classifier information, were stored as explained in Korthals et al. (2017a). The single maps were stitched together, such that they meet the size and resolution of the GT data. Afterward, different combinations of the maps were applied as represented in Table 2 to achieve the corresponding results in the evaluation step.

**Table 2:** Evaluation of static obstacle detection and mapping.

(A) Evaluation A. Process-relevant object detection for single classifiers, classifier combinations, and sensor combinations														

	Single classifiers					Fusion among class					Fusion among sensors			
Classifier	F_1_	Prec.	Rec.	*H*	Fus.	F_1_	Prec.	Rec.	*H*	Fus.	F_1_	Prec.	Rec.	*H*
**Vulnerable Obstacles (Mannequin)**														
cam-LDCF-human	1.3	0.7	25.9	83.2	max.	3.2	1.6	73.4	86.2					
cam-FCN-human	3.4	1.7	73.6	75.6	bay.	12.6	7.1	57.4	84.3					
cam-YOLO-human	11.7	6.9	36.1	75.5										
**Processable (Grass)**														
cam-FCN-grass	85.2	94.2	77.8	75.2										
**Traversable (Grass & Road & Ground)**														
cam-FCN-grass	83.4	96.3	73.6	75.2	max.	84.6	96.0	75.6	75.3	max.	90.1	89.2	91.0	92.3
cam-FCN-ground	24.0	96.8	13.7	75.1	bay.	82.0	97.2	71.0	75.2	bay.	87.7	90.8	84.8	92.2
lidar-SVM-ground	89.7	89.4	90.1	81.1										
**Non-traversable (Vegetation)**														
lidar-SVM-veg.	83.6	81.4	86.0	87.9						max.	84.3	80.1	89.1	92.3
cam-FCN-veg.	46.6	32.2	84.7	81.2						bay.	84.8	81.3	88.7	92.3

**(B) Evaluation B. Traversability assessment of static obstacles for single classifiers, classifier combinations, and sensor combinations**														

	**Single classifiers**				**Bayesian among class**				**Max-pooling among class**					
**Classifier**	**F_1_**	**Prec.**	**Rec.**	***H***	**F_1_**	**Prec.**	**Rec.**	***H***	**F_1_**	**Prec.**	**Rec.**	***H***		

Cam-FCN-human	3.8	25.3	2.1	75.6	13.0	67.4	7.2	89.2	88.8	88.3	89.4	92.5		
Cam-LDCF-human	0.7	3.7	0.4	83.2										
Cam-YOLO-human	1.2	6.8	0.7	75.5										
Radar-tracking	2.6	3.5	2.1	15.9										
Thermal-heatdetection	7.3	16.6	4.7	88.6										
Lidar-SVM-object	7.8	66.8	4.1	89.7										
Cam-FCN-object	4.1	30.8	2.2	76.3	22.3	72.3	13.2	89.5						
Cam-YOLO-object	2.0	3.9	1.3	75.6										
Cam-deepanomaly	2.0	3.8	1.4	75.6										
Radar-tracking	2.6	3.5	2.1	15.9										
Lidar-SVM-object	7.8	66.8	4.1	89.7										
Lidar-SVM-veg.	83.5	81.4	85.8	87.9	84.6	88.3	81.6	92.3						
Cam-FCN-veg.	46.7	32.2	84.4	81.2										

*The vertical lines encapsulate groups of algorithms on the left side and present their fused results on the right hand side. Values in percentages*.

It is worth noticing that the mapping technique is very prone to misclassification, which can be caused for example by sun blinded cameras or systematic errors. To address the second case, a blind spot has been applied at the location of the tractor so that the mapping of self-classification, heavily caused by the radar, was overcome. This approach has been applied to all the following evaluations as well.

The resulting tri-state maps from GT data and mapping were compared tile-wise against each other, such that the true positives (TP), false positives (FP), and false negatives (FN) could be calculated for the entire map.

To do so, the binary mapping *G*: *m* → {0, 1} is defined which converts the cell *m* to an indicator. Furthermore, *G*_GT_ refers to the map constructed from the GT data, and *G*_M_ that maps the cell *m*, given the estimated posterior *P*(*m*) evaluated on the subset of seen cells $M^{\prime }=\left\{m\in M|P\left(m\right)<0.5-\epsilon \vee P\left(m\right)>0.5+\epsilon \right\}$. Thus, *M′* refers to all observed cells which properties are known. To overcome floating-point quantization noise, a slack variable with ϵ=.01 was introduced to the evaluation:

(14) $$\begin{array}{l} G_{\text{M}}\left(m\right)=\left\{\begin{array}{cc} 1, & \text{if}\;P\left(m|z_{1:T},x_{1:T}\right)>0.5\\ 0,\; & \text{otherwise} \end{array}\right.,\;G_{\text{GT}}\left(m\right)=\left\{\begin{array}{cc} 1,\; & \text{if}\;m\;\mathrm{occupied}\\ 0,\; & \text{if}\;m\;\mathrm{unoccupied} \end{array}\right.. \end{array}$$

The function *G*_M_ only takes the estimated map, and *G*_GT_ only takes the GT map into account. TP, FP, and FN can then be calculated by cell-wise multiplication between the estimated map *G*_M_ and the GT map *G*_GT_

(15) $$\text{TP}=\underset{m\in M\prime }{\sum }\;G_{\text{GT}}\left(m\right)G_{\text{M}}\left(m\right)\;,\text{FP}=\underset{m\in M\prime }{\sum }\left(1-G_{\text{GT}}\left(m\right)\right)G_{\text{M}}\left(m\right)\;,\;\text{FN}=\underset{m\in M\prime }{\sum }\;G_{\text{GT}}\left(m\right)\left(1-G_{\text{M}}\left(m\right)\right).$$

The Precision, Recall, F_1_ score, and entropy *H* were calculated as follows:

(16) $$\text{Precision}=\frac{\text{TP}}{\text{FP}+\text{TP}},\;\text{Recall}=\frac{\text{TP}}{\text{FN}+\text{TP}},\;{\text{F}}_{1}=2\frac{\text{Recall}\cdot \text{Precision}}{\text{Recall}+\text{Precision}},$$

(17) $$H\left(P\left(M\right)\right)=-\underset{m\in M}{\sum }\;P\left(m\right)\text{log}P\left(m\right)+\left(1-P\left(m\right)\right)\text{log}\left(1-P\left(m\right)\right)\;.$$

Table 2A shows the results of evaluation **A**, i.e., detecting process-relevant classes exclusively. The results are grouped by the process-relevant classes, and the three columns show individual algorithm detection results, fusion across algorithms, and fusion across sensors, respectively. Here, both competitive (Bayesian) fusion and complementary (max-pooling) fusion were applied for the two fusion scenarios.

Table 2B shows the results of evaluation **B**, i.e., detecting occupied areas with respect to traversability. The first column shows individual detection results for each of the algorithms. These are grouped by object categories such that different algorithms from different sensors that detect similar classes are grouped together. In the second column, algorithms from each group of categories are fused with competitive (Bayesian) fusion. For classifiers detecting the same object classes, competitive fusion increases the precision while maintaining information gain (entropy). In the third column, detections from all sensors (and algorithms) are fused with complementary (max-pooling) fusion. For classifiers detecting different object classes, complementary fusion increases recall while maintaining precision. In practice, this results in a more complete detection of the environment.

Figures 9C–E show an excerpt from the corresponding evaluation in Table 2A. The constructed maps were built from traversing the depicted red track in Figure 8C. The gray area represents unknown or not-seen areas, white denotes a vote for, and black against the desired class. Figure 9C shows the single cam-YOLO-human classification in the top image, whereas the bottom image consists of the combination of all camera-based human classifications. While the single classifier already showed plausible results with correct human classifications highlighted with red circles, it still missed some detections. The combination of classifiers overcame this issue and also increased certainty for classifications where no humans resided. Figure 9D shows the ground and crop classifications of cam-FCN-ground in the top image and the corresponding combination in the bottom. While the camera-based classification showed significant noise at the borders, the classifiers supplemented each other to achieve a denoised and extended classification of the ground. Figure 9E shows the lidar-SVM-vegetation classification in the top image and a combination with camera-based classifiers at the bottom. The lidar already achieved results that were qualitatively close to the GT data. While in the fused result artifacts resulting from the ISM approach are visible at the outer borders, the overall score increased due to the gain of new information and increased certainty of already perceived information. Figure 9F shows an excerpt from the evaluation in Table 2B where a classical retrieval of an occupancy grid map was aimed. The radar classification depicted on the left provided a quite clean obstacle detection which in combination with the remaining object classifiers in the middle led to a richer and more precise result. Finally, the fusion of all classifiers and sensors on the right resulted in a quite complete occupancy map.

### 4.3 Dynamic Scenario

To evaluate the detection of dynamic obstacles, the mapserver was applied in exactly the same way as for the static scenario. However, instead of evaluating a stitched map combining information from traversing the entire field, the mapserver was queried temporally for each available timestamp *t* in the GT data. In order to evaluate only the detection of dynamic and non-static obstacles, recency weighting as introduced in 3.2.4 was applied. The *ForgetValue* and *ForgetRate* are evaluated exhaustively at the end of this section. However, for the following evaluations, a high *ForgetRate* of 6 and a high *ForgetValue* of 0.8 were used, as these values ensured a responsive mapping where only recent measurements were taken into account. In this way, the mapserver continuously updated the positions of moving objects, while still allowing an appropriate amount of information fusion of non-synchronized sensors.

**Figure 10:**
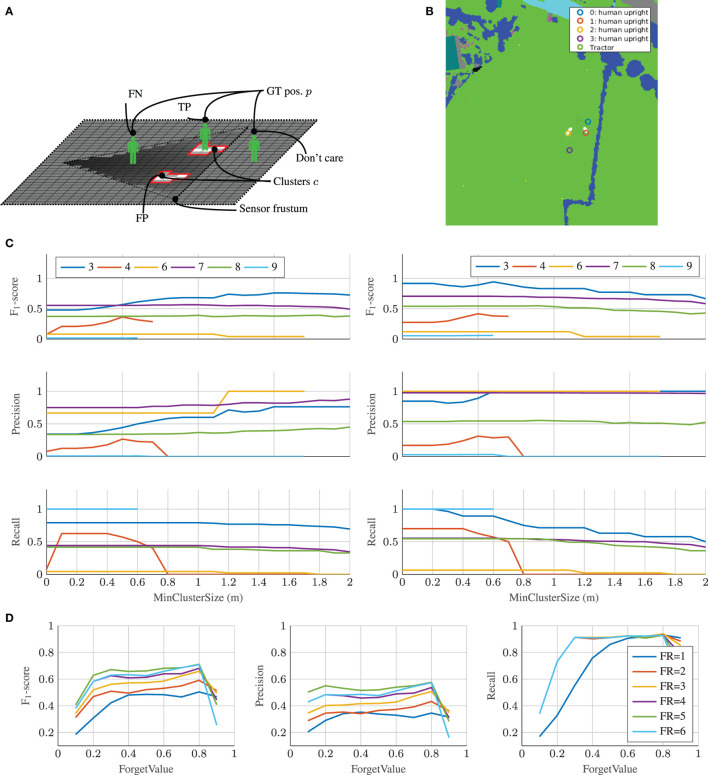
Evaluation of dynamic scenario. **(A)** Dynamic evaluation of TP, FP, and FN acquisition. **(B)** Dynamic evaluation example. Valid clusters are colored white, and GT positions of humans and the tractor are overlaid. **(C)** Precision, recall, and F_1_-score over increasing minimum cluster size for different setups from Table 3. *ForgetRate* = 6 and *ForgetValue* = 0:8. Left: No dilation. Right: Dilation by vehicle radius of 2:5 m. **(D)** F_1_-score over increasing *ForgetValue* with different *ForgetRate* (FR).

Contrary to the static evaluation where GT annotations were dense and pixel-wise, the GT annotations of dynamic obstacles were point-based (Kragh et al., 2017). Therefore, tile-wise comparison between GT data and the fused map was unfeasible. Instead, point-wise GT annotations were compared to clusters of detections for each timestamp. Figure 10A illustrates the dynamic evaluation scenario. First, the different mapserver layers were fused. The resulting tri-state (occupied, unoccupied, unknown) likelihood map was then clustered for each state with 8-connected clustering. Clusters smaller than *MinClusterSize* were pruned to suppress noise. Finally, TP, FP, and FN were accumulated over time in the GT data by comparing the detected clusters *c_j_* with index *j* and the GT positions *p_i_* with index *i*:

(18) $$\begin{array}{lll} \text{TP}=\underset{t}{\sum }\;{\text{TP}}_{t},\;{\text{TP}}_{t}=\left|\left\{p_{i}|\exists c_{j}:p_{i}\in c_{j}\right\}\right|,\; & & \;\\ \text{FP}=\underset{t}{\sum }\;{\text{FP}}_{t},\;{\text{FP}}_{t}=\left|\left\{c_{j}|p_{i}\notin c_{j}\right\}\right|,\; & & \;\\ \text{FN}=\underset{t}{\sum }\;{\text{FN}}_{t},\;{\text{FN}}_{t}=\left|\left\{p_{i}|p_{i}\notin c_{j}\right\}\right|. \end{array}$$

Regions that remained unknown (*P*(*m*) = 0.5) did not affect the evaluation, and only detected clusters and GT positions inside the sensor frustum were taken into account. Similar to the static scenario, precision, recall, and F_1_-score metrics were calculated using equation (17). Figure 10B shows an example from the dynamic evaluation. The GT positions are denoted by colored circles, while the detected clusters are represented by white regions beneath. In the depicted example, one true-positive, one false-positive, and one false-negative were counted due to the fact that the yellow and red positions were inside the sensors’ frustum.

Figure 9B illustrates the evaluation pipeline for the temporal sequences. In an offline-procedure, all necessary GT information like person identifiers (ID), their status (visible/non-visible and standing/sitting/lying), the geo-referenced locations, and the timestamps was extracted. Afterward, the mapserver ran in a common setup with the forgetting feature, where for every given GT timestamp the current maps of the mapserver were extracted. In an evaluation step, the maps were clustered and compared to the GT information to achieve the presented results in Figures 10C,D.

Table 3 lists 9 different sensor/algorithm setups that were evaluated. Setup 1 includes all sensors and algorithms, setup 2 includes all stereo camera algorithms, whereas setup 3–9 concern individual sensors and detection algorithms.

**Table 3:** Listing of setups and the detection algorithms they comprise.

Class	Object	Heat	Object	Objects/human	Human	Human	Anomaly
Algorithm	Detection	DynamicHeat	SVM	FCN	LDCF	YOLO	DeepAnomaly
	9 (Radar)	3 (IR)	4 (Lidar)	5	6	7	8
	
Setup				2 (Camera)			
	
	1						

Figure 10C shows precision, recall, and F_1_-scores for setup 3–9, when varying the *MinClusterSize* used in the clustering. Figure 10C (right) shows results for clustering without subsequent dilation, whereas Figure 10C (left) introduces dilation by the vehicle radius of all clusters as is common in robotic navigation and planning algorithms (Dudek and Jenkin, 2010). In the current evaluation, dilation effectively mitigated the influence of localization inaccuracies and resulted in better scores. Objects that were detected and mapped with slight displacements from their GT positions were thus more likely to be included by dilated clusters. This indicated that a large part of false-negative detections were located close to GT positions. Setup 4 (lidar) and 9 (radar) had undefined F_1_-scores for *MinClusterSize*s above 0.7 and 0.6 m, respectively. This was caused by the two sensors providing precise 3D measurements, which made their detections precisely located and narrow in space. Since the human objects had small footprints, no clusters with areas above these values were generated. For the same reason, a *MinClusterSize* of 0.5 m was chosen as a compromise, such that most of the noisy sensor readings were filtered out, while small and correct detection footprints from humans were still kept.

Table 4 shows precision, recall, and F_1_-scores for the fusion setups 1 and 2 using *MinClusterSize* = 0.5 and no subsequent cluster dilation. For setup 1 (all sensors and algorithms), complementary (max-pooling) fusion performed much better than competitive fusion. This was caused by the fact that detections from different sensors did not overlap perfectly due to localization errors. Competitive fusion, therefore, falsely combined non-overlapping detections, whereas the complementary fusion tolerated the localization issues by effectively summing all detection contributions. For setup 2 (camera-based detection), however, competitive (Bayesian) fusion was superior to complementary fusion. This was caused by the fact that the same camera was used by all algorithms, thereby mitigating localization errors and ensuring overlapping detections.

**Table 4:** Sensor fusion of setup 1 and 2 with different fusion strategies.

Setup	Fusion	F_1_ (%)	Precision (%)	Recall (%)
1	Max	70.81	57.23	92.86
	Bayes	42.58	39.76	45.83
2	Max	57.32	51.14	65.22
	Bayes	61.22	56.96	66.18

Figure 10D shows precision, recall, and F_1_ scores for setup 1 (all sensors), when varying the *ForgetRate* (1–6) and *ForgetValue* (0.1–0.9) of the mapserver. Similar to the above cases, *MinClusterSize* = 0.5 and no subsequent cluster dilation was applied. Clearly, all scores were dramatically influenced by the two parameters. A *ForgetValue* of 0.8 and *ForgetRate* of 6 seemed to be the best compromise between memory and responsiveness, such that only the most recent measurements were taken into account. A too large *ForgetValue* (close to 1) resulted in no memory, meaning that valuable information from previous frames was not taken into account. Contrarily, a too small *ForgetValue* (close to 0) resulted in too long memory (approaching the static scenario), effectively letting outdated information of obstacle positions stay in the map. Similarly, a too small *ForgetRate* resulted in too long memory, whereas the performance seemed to approach an upper limit with larger *ForgetRate*s.

### 4.4 Process Evaluation

The above-mentioned approaches were able to classify single observations point-wise and did not take into account surrounding classifications when determining classes of current observations. In agricultural processes, however, observations obtained from the surroundings are typically identical and homogeneous in particular. Furthermore, certain transitions between classes are rather unlikely. For example, if the classification of the current pose is *Grass*, it is rather unlikely that the classification of the next pose is *Ground*. From the processable class *Grass* to the traversable class *Road*, there are commonly ground, borders, or trenches. To utilize these dependencies between the individual classes, we use a Hidden Markov Model (HMM) and calculate the belief P(O,w;λ) about the class model λ from equation 11. The observation per pose is the feature vector from equation (6) of homogeneous clusters extracted via SEVDS (see equation (5)). The sequence of observations along some trajectory contains the upcoming poses of the vehicle as depicted in Figure 7A. A consequence of having metric grid maps is that via the shape constraints in equation 5, implicit sizes of clusters can be given. This influences the step size of every pose to decode along the trajectory, so that empirically shape parameters for given step sizes can be found.

To compare the capabilities of the HMM against the static scenario from subsection 4.2, the same process-relevant classes (denoted in brackets) were chosen to train four different models (*I* : = 4 w.r.t. equation (11)): Vulnerable Obstacles (Mannequin), Processable (Grass), Traversable (Ground), and Non-Traversable (Vegetation). It is worth mentioning that the class *Grass* was removed from the model *Traversable* to make it mutually exclusive against the model *Processable*.

**Figure 11:**
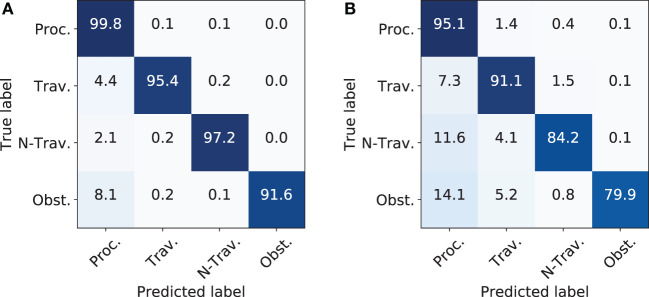
Results for decoding the corresponding classes along the trajectory at close and far range. **(A)** Confusion matrix for near field. **(B)** Confusion matrix for far field.

The entire training was performed in a supervised fashion on the mapped data from the static scenario. All inter-property models as depicted in 7c had five hidden states (*J* : = 5 w.r.t. Equation 11) due to the fact, that less states result in worse performance and more states do not show any improvements. The minimum amount of states can be explained by the necessary modeling of the burn-in and out behaviors as stated in 3.4.2, while more states do not improve the performance as the models tends to exit after the fifth state. Furthermore, the training set was extracted out of randomly generated trajectories, while the test set represented trajectories driven by the vehicle. It was desired to forecast the class along the trajectory for as long as possible, but the maximum length was constrained by two factors: First, the applied mapserver only had a locally bounded area, where the maximum allowed range reading was equal to the size of the outer boundary minus the inner boundary (Kragh et al., 2016). For the presented experiments, the boundaries were set to 35 and 10 m, respectively, which resulted in a maximum forecast of 25 m. Second, not all sensors exploited this maximum range reading and further, closer areas tended to be more precise in information due to the nature of the occupancy grid mapping algorithm. Thus, to have a fair comparison, the decoding was done for a close range starting from the tractor at 0 to 12.5 m (Figure 11A) and a far range extending the former range from 12.5 to 25 m (Figure 11B).

For training, the HMM was initialized as follows: all start and property probabilities were uniformly distributed with an additive Gaussian noise. The transmission probabilities of the property models were randomly initialized. The beta distribution mean for emission was set by k-means++ (Arthur and Vassilvitskii, 2007), while the variance was kept constant. Training and decoding was performed on all available detection algorithms as presented in Table 2B.

## 5 Discussion

The proposed architecture is an extension of the authors’ previous paper on occupancy grid mapping in agriculture (Kragh et al., 2016). The current study has unified the system architecture and extended the previous approach by a class-specific evaluation of static obstacles plus a method for detecting and mapping dynamic obstacles over time. Furthermore, this paper has introduced a process evaluation method combining mapped environment detections over time into agriculturally relevant properties.

The provided evaluation measured the end-to-end ability of both fused and individual algorithms to detect and map elements with the provided architecture. That is, detections were not evaluated in local sensor frames, but were instead evaluated after projection to local 2D grids and after global mapping. A deficiency of such an evaluation was that it did not clarify why a given algorithm or sensor performed badly. The end-to-end detection error may have originated from multiple sources, such as sensor noise, detection or local localization errors by algorithms, errors in intrinsic and extrinsic calibration parameters, inaccurate grid map representations, robot localization errors, and errors in the ground truth annotations. To isolate and quantify these error sources, GT data would be necessary for each link in the chain. However, annotations of obstacles were only available as global GPS-coordinates and not in the local vehicle frame or sensor frames (e.g., pixel-wise or bounding box annotation in camera images).

After fusing all sensors, the complete architecture reached an F_1_-score of 88.8% in static traversability assessment (Table 2B) and 70.8% in dynamic obstacle detection (Table 4). The presented performance measures are useful for showing relative improvement with fusion and for comparing proposed methods. The metrics, however, cannot quantify the safety-level of the system in real operation. A very low F_1_-score for e.g., camera-based human detectors in Tables 2A,B suggests that the combined localization and detection is of insufficient performance. However, an actual safety system should not be evaluated on F_1_-scores of a map, but instead on, e.g., the decoded process-relevant properties along the traversed trajectory as in Figure 11. As of today, no self-driving cars are certified for full autonomy, and, to the authors knowledge, no regulations describe exactly what detection accuracy, precision, frequency, etc. would be required for certification. Instead, self-driving car manufacturers document their traveled distances during testing without incidents and without human intervention. An actual certification might end up building on measures like these. And most likely, autonomous vehicles in agriculture will follow and possibly extend the regulations of self-driving cars, once available.

As shown in Tables 2A,B, classification performance generally increased as more sensors were introduced. However, different sensors detecting the same class may not always lead to a significant increase in accuracy. In fact, this was the case for the radar. The fusion of all sensors in Table 2B gave an F_1_-score of 88.873%. The same setup without the radar gave an F_1_-score of 88.871%, which was hardly a significant improvement. The specific radar and detection algorithm pair could, thus, be left out of the fusion setup, as it did not contribute with more information. On the other hand, even with insignificant improvements, additional sensors may still provide a more robust and redundant setup, thus mitigating single points of failure. And with another radar, specifically targeting agricultural scenarios (e.g., by penetrating vegetation), actual improvements in accuracy may be possible.

The results in Figure 10C (right) showed that the F_1_-score could be improved significantly by introducing a cluster dilation corresponding to the vehicle size in the dynamic evaluation. Effectively, the dilation mitigated the influence of localization and demonstrated the potential of the detectors when being less sensitive to localization errors. An optimized localization, a model-based approach, or temporal tracking of detected clusters would, therefore, potentially increase the combined detection and localization results.

As previously mentioned, localization errors could also originate from inaccurate grid map representations in the ISMs. This could be caused by extrinsic and intrinsic calibration errors for each sensor, such that detections in the local sensor-frames were incorrectly transformed to the vehicle-frame.

Multiple heuristic models were introduced in the ISM to convert detections into occupancy probability estimates. Heuristic model parameters have been selected to model both detection and localization uncertainties for a given algorithm. In future work, these issues could be addressed by supervised training of a function approximator for mapping detections from local sensor-frames to the vehicle-frame as well as converting detection certainties to occupancy probabilities. Effectively, this could limit the number of heuristics and improve both localization and detection accuracy. One example is the heuristic model used for converting 2D bounding boxes to an OGM using a stereo camera as explained in paragraph 3.3.1.2. The uncertainty for localizing an object is modeled using assumed radial and the angular variances. However, the true radial and angular variances can be estimated more accurately from sensor calibrations. A more extensive approach would be to train the ISMs end-to-end, such that environment detections were directly output in local vehicle coordinates. However, this would contradict our applicable architecture approach that allows easy setup of different sensor combinations and would require a much larger dataset for training.

The semantical occupancy mapping technique used competitive (Bayesian) fusion for similar modalities followed by complementary (max-pooling) fusion for dissimilar modalities. This was both an intuitive and a reasonable procedure for fusing information and was demonstrated to increase the F_1_-score. More advanced procedures such as instance boosting could be trained to learn the optimal combination of semantical maps. Such procedures would expectedly be less prone to misclassifications such as cameras blinded by the sun or potential systematic errors. Comprising the three possible levels of fusion, which are fusion on raw data, feature level, or decision level, our approach focuses on decision level fusion. Other approaches like Kalman filtering techniques tend to work on raw data and at feature level which might result in better fusion results, but also demand more effort in designing the filters themselves. Furthermore, varying setups cannot be considered easily due to necessary redesign of the filter. On the other hand, model-free approaches like particle filters have proven their capabilities also for occupancy grid map approaches by Korthals et al. (2016), but would need deeper insights into the sensors’ design to build up proper fusion. As stated before, this approach pursues the easy changeability and extendability of sensors and other information sources. Considering this condition, the occupancy grid mapping technique tends to be the most versatile approach, which allows the combination and incorporation of information also after the sensor data has been mapped.

Process evaluation was implemented to be executed at runtime. The Hidden Markov Model (HMM) was applied to decode the most recent SOGM along the upcoming vehicle trajectory. This approach allowed the process evaluation along a vehicle trajectory to predict and steer machine parameters for upcoming situations. The training and decoding was performed such that intra-property-HMMs modeled the process-relevant classes for a grass mowing scenario which were linked together in a inter-property-HMM. Results showed a detection rate of over 90% for every class in near-field situations, whereas the detection rate degraded noticeably in far-field situations. The drop performance can be explained by the map-building process. Far-field areas have only been observed a few times and are, therefore, prone to classification errors. Near-field areas have been observed more often and are less sensitive to similar noise. Furthermore, detection algorithms are expected to perform better at short range. Thus, the proposed HMM approach for combining the classifications inside the SOGM has proven its capabilities to learn the process’s statistics and correct combination of SOGMs to predict the correct classes. Other approaches such as boosting are applicable for classifier fusion as well. However, the structure of HMM is better suited for modeling the statistics and consecutiveness of the given processes.

However, with our proposed architecture pipeline and information processing we have shown that with each combination of classifiers, an overall increase of the F_1_-score can be reached. With up to 88.8% in a 10 cm cell-wise, globally mapped evaluation for obstacle scenarios, our approach represents a state-of-the-art solution for environment classification in agricultural scenarios. Similar results were achieved for mapping semantical classes so that further mowing processes can be prospectively controlled by this information. Finally, the proposed application of HMMs to decode process-relevant information directly from the SOGMs has shown that our architecture is online applicable.

## 6 Conclusion and Future Work

In this work, we have presented an information processing architecture for global mapping and process evaluation in an agricultural grass mowing scenario. The proposed architecture consists of four components: Sensor Platform, Inverse Sensor Models, Fusion and Mapping, and Process Evaluation. The sensor platform comprises all applied sensors for localization and environment data acquisition, such as stereo camera, radar, lidar, and thermal camera. The inverse sensor models (ISMs) describe the sensors’ data processing for detecting and localizing process-relevant properties and objects in the environment, such as grass, vegetation, and humans. The ISMs are 2D grid-based, non-parametric representations of the detection outputs. Fusion and mapping is performed on the ISMs which are referenced and fused based on the occupancy grid mapping algorithm into a semantical occupancy grid map (SOGM) stack. Process evaluation applies a Hidden Markov model-based approach to first train and then quantify the environment along the vehicle’s trajectory to reveal process-relevant information out of the SOGMs.

To evaluate the capabilities of the mapping approach, we compared the mapping and fusion of ISMs in a static and dynamic obstacle scenario against the FieldSAFE dataset. For both scenarios, we reported detection results for individual classifiers, fusion among classifiers, and fusion among sensors. In the static case, detection and localization results improved when introducing information fusion, first through competitive fusion among classifiers detecting similar classes, and second through complementary fusion among sensors and algorithms detecting different classes. For detecting humans in the dynamic evaluation, only classifiers that were able to detect these were fused accordingly, before a grid cell clustering was applied to retrieve consistent human hypotheses. Furthermore, the SOGM method was extended with forgetting capabilities to adapt the mapping approach to dynamic environments. Similar to the static evaluation, a combination of multiple sensors led to an overall improvement in detection of dynamic obstacles.

In future work, we want to incorporate geodata acquired by satellites, drones, or planes from which we directly derive process-relevant information into the detection pipeline. This approach will overcome issues such as complex sensor registration, weather conditions, and false detections for static properties and objects in the environment, and will, therefore, improve and harden our setup. Furthermore, we want to apply supervised training of the mapping from sensor-frames to the vehicle-frame for ISMs, thereby reducing heuristics and improving global localization.

## Author Contributions

The overall contribution and workload have been distributed between TK, MK, and PC in a close collaboration. TK is responsible for subjects related to fusion, mapping, process evaluation, and for evaluating the ability of the whole architecture to detect static and dynamic obstacles and perform process evaluation. MK and PC designed and implemented detection algorithms and inverse sensor models. RJ contributed with insight into the domain of agriculture and preparing field experiments. HK contributed with insight into machine learning and detection algorithms. UR contributed with insight into robotics and systems engineering.

## Conflict of Interest Statement

The authors declare that the research was conducted in the absence of any commercial or financial relationships that could be construed as a potential conflict of interest. The reviewer, AP, and handling editor declared their shared affiliation.

## Footnotes

^1^The sensor frustum is the perceptible volume of a sensor, also referred to as the field of view or lobe.

^2^https://vision.eng.au.dk/fieldsafe/ (Accessed: March 15, 2018).

^3^Global Navigation Satellite System.

^4^ROS package available at https://github.com/PeteHeine/pedestrian_detector_ros.git (Accessed: March 15, 2018).

^5^ROS package available at https://github.com/PeteHeine/yolo_v2_ros (Accessed: March 15, 2018).

^6^Model is available at https://github.com/shelhamer/fcn.berkeleyvision.org (Accessed: March 15, 2018).

^7^ROS package available at https://github.com/PeteHeine/fcn8_ros (Accessed: March 15, 2018).

^8^ROS package available at https://github.com/PeteHeine/dynamic_heat_detection (Accessed: March 15, 2018).

^9^ROS package available at https://github.com/PeteHeine/image_inverse_sensor_model2 (Accessed: March 15, 2018).

^10^ROS package available at https://github.com/PeteHeine/image_boundingbox_to_3d (Accessed: March 15, 2018).
